# Olaparib in HR-deficient, metastatic triple-negative breast and platinum-sensitive relapsed ovarian cancers without germline mutations in *BRCA1/2*: phase 2 EMBRACE trial

**DOI:** 10.1038/s41416-026-03535-6

**Published:** 2026-07-08

**Authors:** Katrin M. Sjoquist, Alexander Dobrovic, Kristy P. Robledo, Ksenija Nesic, Sally Baron-Hay, Sumitra Ananda, Nicole McCarthy, Jeffrey Goh, Christopher Steer, Nick Murray, Sonia Yip, Linda R. Mileshkin, J. Lynn Fink, Garry Chang, Lisa Bailey, Yvonne Lee, Danka S. Zebic, U. G. Imalki U. Kariyawasam, Kristy Shield-Artin, Andrew Jarratt, Justin Bedő, Anthony T. Papenfuss, Olga Kondrashova, Matthew J. Wakefield, Cassandra J. Vandenberg, Martin R. Stockler, Clare L. Scott, Paul M. Waring

**Affiliations:** 1 NHMRC Clinical Trials Centre, The University of Sydney, Camperdown, Australia; 2 Cancer Care Centre, St George Hospital, Kogarah, Australia; 3 Australia New Zealand Gynaecological Oncology Group, Sydney, Australia; 4 Department of Surgery, The University of Melbourne, Parkville, Australia; 5 Walter and Eliza Hall Institute of Medical Research, Melbourne, Australia; 6 Department of Medical Biology, University of Melbourne, Melbourne, Australia; 7 Royal North Shore Hospital, Sydney, Australia; 8 Breast Cancer Trials ANZ, Newcastle, Australia; 9 Peter MacCallum Cancer Centre, Melbourne, Australia; 10 ICON Cancer Centre Wesley Auchenflower, Auchenflower, Australia; 11 Royal Brisbane & Women’s Hospital, Herston, Australia; 12 Border Medical Oncology Research Unit, Albury Wodonga Regional Cancer Centre, Albury, Australia; 13 UNSW School of Clinical Medicine, Rural Clinical Campus, Albury, Australia; 14 Royal Adelaide Hospital, Adelaide, South Australia; 15 Sir Peter MacCallum Department of Oncology, University of Melbourne, Melbourne, Australia; 16 XING Genomic Services, Brisbane, Australia; 17 Department of Medical Oncology, Wollongong Hospital, Wollongong, NSW Australia; 18 QIMR Berghofer Medical Research Institute, Brisbane, Australia; 19 Department of Obstetrics, Gynaecology and Newborn Health, University of Melbourne, Melbourne, Australia; 20 Department of Translational Pathology, AstraZeneca, Cambridge, UK

**Keywords:** Ovarian cancer, Breast cancer, Tumour biomarkers, Cancer genomics

## Abstract

**Background:**

Homologous recombination (HR) deficiency (HRD) from germline *BRCA1/2* mutations (gBRCAm) sensitizes high-grade serous ovarian cancer (HGSOC) and triple-negative breast cancer (TNBC) to PARP inhibitors (PARPi), as may promoter methylation of *BRCA1* (me*BRCA1*) or *RAD51C* (me*RAD51C*) or mutation of non-BRCA HR genes. This trial evaluated olaparib in platinum-sensitive, relapsed HGSOC (PSROC) and metastatic TNBC (mTNBC) with non-gBRCA HRD.

**Methods:**

Single-arm phase 2 trial of olaparib 300 mg orally twice daily. Tumour me*BRCA1/*me*RAD51C* determined by methylation-sensitive, high-resolution melting PCR and targeted sequencing. Unmethylated cases underwent HR gene mutation testing. Primary outcome: objective tumour response rate (OTRR) at 6 months(m). Secondary outcomes: progression-free survival (PFS), OTRR according to HR gene aberration, and safety.

**Results:**

Total 22 enrolled: promoter methylation detected in 8/15 HGSOC and 5/7 TNBC, and pathogenic variants (PV) in non-*BRCA* HR genes. OTRR at 6 m was 40% HGSOC and 0% TNBC. OTRR was 38% in methylated cases vs 43% for other HRD. 6 m/12 m PFS were 53% / 25% (HGSOC), and 17% / 0% (TNBC).

**Conclusions:**

Olaparib demonstrated activity in HGSOC beyond gBRCAm, including with me*BRCA1* or *gRAD51C* PV. Olaparib had limited activity in pre-treated TNBC. Effects of prior chemotherapy on me*BRCA1/*me*RAD51C* require exploration to improve patient selection for PARPi.

**Clinical trials registration:**

Australian New Zealand Clinical Trials Registry Registration number ACTRN 12617000855325.

## Background

Despite current best treatments, advanced breast and ovarian cancers remain a significant clinical challenge. Better treatment options are needed to improve survival of women with relapsed high-grade serous ovarian cancer (HGSOC) and metastatic triple negative (ER-, PR-, HER2-) breast cancer (TNBC). Poly(ADP-ribose) polymerase inhibitors (PARPi), such as olaparib, have been in clinical use for nearly a decade, with improved outcomes in selected patients with breast [1, 2] and ovarian cancers in patients with germline *BRCA1/2* pathogenic variants (PV) (gBRCAm) [3] and prostate cancers harbouring germline or somatic PV in genes involved in homologous recombination (HR) DNA repair (HR genes) [4]. With increasing evidence for efficacy of PARPi in HR deficient (HRD) breast, ovarian and prostate cancers without gBRCAm, there is an urgent need to improve understanding of mechanisms of HR deficiency and optimise patient selection [5, 6].

Mechanisms of HRD other than gBRCAm occur in HGSOC and TNBC, with evidence of PARPi activity in cases with wildtype *BRCA1/2* (BRCAwt) and high genomic HRD scores [3, 7, 8]. Somatic BRCAm (sBRCAm) in the absence of a gBRCAm, confers sensitivity to PARPi in rare subsets of TNBC (3.2−5%) [9] and HGSOC (1.6–4%) [8, 10, 11]. Germline and somatic PV in non-*BRCA1/2* HR genes, collectively account for another 6% and 3% of ovarian cancer [10] and 3.7% and an unknown number of breast cancer [12], respectively. Patients with g*RAD51C* and g*RAD51D* PV have indistinguishable survival outcomes to patients with gBRCAm, indicating that pathogenic HR pathway mutations appear to be clinically equivalent [13].

Other mechanisms resulting in HRD include epigenetic silencing of *BRCA1* (me*BRCA1*) or *RAD51C* (me*RAD51C*) by promoter hypermethylation [5, 14–16] Although accurate population-based prevalence data is lacking, epigenetic silencing of *BRCA1* has been reported to occur in 15% of sporadic breast and ovarian cancers, and is enriched in TNBC (29−46%, compared to 5% of ER + /PR+ and 2% of HER2 + ) and HGSOC (10−20%) [17–19]. Me*BRCA1* and gBRCAm are mutually exclusive, suggesting that either is sufficient to cause loss of function [17, 20]. Moreover, *gBRCA1* and me*BRCA1* confer a similar prognosis in TNBC [11, 21] and share common morphological features, including an increase in tumour infiltrating lymphocytes (TILs)[20, 22], suggesting they exert similar oncogenic and clinical effects. Collectively, germline, somatic and epigenetic changes in the HR pathway account for 56–73% of TNBC and 42–49% of HGSOC [7, 14].

Early studies retrospectively evaluating the efficacy of PARPi in patients with me*BRCA1* ovarian cancer produced conflicting and inconclusive results, likely due to use of non-quantitative methylation assays [21]. We and others subsequently demonstrated the importance of distinguishing homozygous (biallelic) from heterozygous (monoallelic) and homogeneous (all CpG sites methylated) from heterogeneous (some GpG sites methylated) promoter methylation [23] and the need to quantitate methylation [24] as well as adjust raw methylation measurements by tumour cellularity and gene copy number [25]. Being tumour suppressor genes, all copies present (including if > 2 copies) of *BRCA1* or *RAD51C* must be fully methylated to result in transcriptional silencing, loss of protein expression, loss of RAD51 foci formation, HR deficiency, and to confer both platinum and PARPi sensitivity [25, 26].

Similar to the concept of the selective acquisition of PARPi-resistant secondary mutations in OC/TNBC cells with a primary HR gene mutation [27, 28], promoter methylation is potentially even more dynamic in response to selective pressures, such as prior treatment. In HGSOC, homozygous me*BRCA1* predicts response to PARPi [29], while loss of promoter methylation of just one allele of *BRCA1* is sufficient for loss of sensitivity to both platinum and PARPi [25, 27]. The role of me*BRCA1* in breast cancer, however, is less clear, with similar technical issues hampering earlier research and the lack of conclusive evidence of benefit from PARPi because patients tested had been heavily pre-treated driving loss of silencing [30]. Recent work accounting for the proportion of me*BRCA1* suggests activity of PARPi in TNBC [31] with high but not low me*BRCA1*[32]. The role of me*RAD51C* as a driver of HRD is well-described in HGSOC [33, 34], with some evidence of PARPi response when homozygously methylated, and may be relevant for TNBC given its association with gene silencing and HRD signatures [35]

Increasing exposure to platinum chemotherapy is associated with loss of me*BRCA1* and resistance to PARPi in HGSOC [29]. Differences in epigenetic signatures between paired primary and metastatic HGSOC [29] or TNBC in the AURORA screening initiative [36], indicate that methylation is reversible and may change during the course of the disease [36]. There is also anecdotal evidence that patients who maintain biallelic me*BRCA1* or me*RAD51*, despite selective treatment pressure, can experience exquisite [37] and /or long-term durable responses to PARPi [29].

When this trial was conceived, no prospective studies had demonstrated activity of PARPi in TNBC or HGSOC found to be HRD due to specific genetic or epigenetic changes other than by gBRCAm. Given a strong biological rationale for activity in other HRD cancer types [7, 14, 38–41], the aim of this proof-of-concept study was to determine the activity of olaparib in patients with metastatic TNBC or relapsed HGSOC whose tumours were HRD due to specific genetic or epigenetic changes, other than by gBRCAm.

*BRCA1* methylated subjects were deemed eligible for enrolment if methylation was detected above the analytical limit of detection of the pre-screening assay. However, during the conduct of this trial, the role of me*RAD51C* [33], the importance of -quantifying and adjusting the proportion of methylation for tumour cell content and ploidy [14, 24, 25] and the discovery of methylation loss as a resistance mechanism [30, 36] all became apparent, resulting in the later inclusion of me*RAD51C* participants, the development and retrospective use of a more quantitative me*BRCA1* and me*RAD51C* next generation sequencing (NGS) assay [42] and ethics approval to undertake additional retrospective correlative studies, including whole exome sequencing (WES) to better measure tumour purity and gene copy number (CN) and to investigate potential mechanisms of resistance.

This trial was an essential step towards improving therapeutic options for this group of under-served patients and in developing a clinically deployable assay to quantitate me*BRCA1* and me*RAD51C* for evaluation in future clinical trials.

## Methods

### Study design

This open-label, single-arm, multi-centre phase II trial (EMBRACE) assessed the activity of olaparib as a single agent in two molecularly enriched cohorts. The key eligibility criteria were participants aged ≥18, ECOG 0–1, who had measurable disease by RECIST v1.1, and adequate bone marrow, renal and liver function, with an available HRD screening result showing HRD without germline *BRCA1/2* mutations with either [1] metastatic TNBC or [2] relapsed platinum-sensitive HGSOC. Participants in the TNBC cohort must have received anthracycline and/or taxane-containing chemotherapy, and in the HGSOC cohort must have received a single line of platinum-containing chemotherapy (adjuvant or first line metastatic) only. More than one prior line of platinum-based chemotherapy was allowed for participants with HGSOC containing germline mutations in non-*BRCA* genes given that no evidence existed at the time, in this group, of acquired resistance with multiple lines of platinum-containing therapy. For the purposes of recruitment, HRD was defined as presence of somatic or germline likely pathogenic/pathogenic variant (PV) in HR gene or presence of promoter methylation of *BRCA1* or *RAD51C*. All participants provided written informed consent prior to study procedures. The protocol and all amendments were approved by the Sydney Local Health District Human Research Ethics Committee and local institutions in all participating centres. Australian New Zealand Clinical Trials Registry Registration number ACTRN 12617000855325.

We aimed to recruit patients with somatic *BRCA1/2* mutations, somatic or germline likely pathogenic/pathogenic variants (PV) in other HR genes, or promoter methylation of *BRCA1*, and later of *RAD51C*. To avoid over-representation of somatic *BRCA1/2* cases, recruitment of patients with somatic *BRCA1/2* mutations to the HGSOC cohort was to be capped at eight participants. Additionally, two patients with either HRD mutational signature 3 [43] assessed using targeted Comprehensive Cancer Panel (Agilent Design ID 3016871) or HRD-mean score genomic scar [44] assessed through INNOVATE program [45] were also included.

Screening activities were paused for two periods during 2020-2021 due to COVID restrictions, and briefly in 2019 due to a change in the provider for pre-screening activities. Recruitment was closed early due to slow accrual and funding limitations

### Pre-Screening for non-*gBRCA* HRD

A pre-study screening test of blood and formalin-fixed paraffin-embedded (FFPE) tumour tissues was performed on all potential participants who consented to the pre-screening program. The test was conducted to identify somatic *BRCA1/2* PVs, *BRCA1* or *RAD51C* promoter methylation, somatic or germline PVs in other HR genes, and to exclude *gBRCA1/2* PVs as the mechanism of HRD. All pre-screening tests were performed in a ISO15189 certified diagnostic laboratory using validated and accredited assays Specimens were preferred to be the most recent obtained, ideally after any previous (neo-adjuvant) treatment. Re-biopsy was encouraged but not mandated. Tumour promoter methylation was determined, initially for me*BRCA1* alone and later also for me*RAD51C*, by semi-quantitative methylation-sensitive high-resolution melting (MS-HRM) PCR [46–48]. Since *BRCA1* and *RAD51*C promoter methylation appeared to be either present or absent, patients were enrolled if there was >4% methylation of either promoter, which was the analytical limit of detection of the MS-HRM assay. Later, samples from all enrolled subject were re-tested using the Oncoreveal^TM^ *BRCA1* and *RAD51C* methylation panel (Pillar Biosciences Natick, MA), [42, 49] to accurately quantitate the level of *BRCA1* and *RAD51C* methylation [25].

Unmethylated cases then underwent paired tumour - blood NGS HR gene mutation testing using Oncoreveal^TM^ HRDv2 Panel (Pillar Biosciences). The HRD panel comprises 33 genes including 22 HR-associated genes (*BRCA1, BRCA2, ATM, ATR, BARD1, BRIP1, CDK12, CHEK2, FANCA, FANCD2, FANCE, FANCF, MRE11A, NBN, PALB2, PPP2RIA, RAD50, RAD51, RAD51B, RAD51C, RAD51D, RAD54L*) plus 11 other genes to provide molecular confirmation of the cancer subtype, including *TP53*. The PV allele frequency (VAF) of mutant *TP53* can be relied on in HGSOC and TNBC as a surrogate for tumour cellularity and a measure of tumour purity. Only patients with a PV (pathogenic or likely pathogenic, Class I or II), either germline (other than *gBRCA1* or *gBRCA2*), or somatic, in any of the 22 HR genes or detectable *BRCA1* or *RAD51C* promoter methylation were eligible for enrolment. Cases with HR variants of uncertain significance (VUS) or g*BRCA1/2* mutations determined from the blood sample were excluded from the study. Patients with me*BRCA1* or me*RAD51C* cancers were initially deemed to be *BRCA1/2* wild-type due to known mutual exclusivity of germline and methylated cases, and this was later confirmed with NGS testing for all enrolled subjects and WES for the majority of enrolled subjects, following study completion. Post-enrolment WES and HRD genomic scar scoring was performed as previously described [50](Supplementary Methods, Supplementary Table 5).

### Treatment and study assessments

All participants received olaparib 300 mg twice daily, until disease progression or prohibitive toxicity. Clinical evaluation including relevant tumour markers (Ca-125/CA15-3) and computer tomography (CT) scan were performed at baseline, followed by clinical assessment including blood tests every 4 weeks, and 8-weekly CT scans until disease progression. Adverse event (AE) assessment was performed every 4 weeks and approximately 30 days after the last dose of study treatment and were classified according to the CTCAE v.4.03. Bloods (optional) were collected for translational research at baseline, day 1 of cycles 2 and 5, end of treatment and/or at disease progression. Biopsies for research (optional) were conducted at pre- and post-study treatment.

### Outcomes

The primary outcome was objective tumour response rate (OTRR), defined as the proportion of evaluable participants with confirmed complete response (CR) or partial response (PR) by 6 months according to RECIST v.1.1 in each tumour cohort (note that patients who were deemed to have a response status by 6 months, and the response was subsequently confirmed after 6 months, were included in the OTRR). Evaluable patients were defined as patients who received at least one cycle of therapy and had their disease re-evaluated for response. Central quality review of radiology reports was performed for the primary outcome.

Secondary outcomes were PFS, OTRR according to HR gene aberration (promoter methylation or non-methylated due to other cause of HRD), adverse events, and activity of olaparib in the HGSOC cohort as determined by the CA-125 response according to GCIG criteria [51]; at least 50% reduction in CA-125 levels from the baseline sample and confirmed and maintained for at least 28 days. A post hoc analysis of the duration of the OTRR was also performed.

### Sample size

Using two-stage Simon design (Minimax), a total of 25 TNBC and 28 HGSOC participants would provide 80% power and 95% confidence to exclude response rates of 10% and 15% in favour of an informative rate of 30% and 35% for TNBC and HGSOC, respectively. Following enrolment of 15 patients in each cohort in the first stage, progression to the second stage with further enrolment of 10 and 13 patients for the TNBC and HGSOC cohorts respectively was dependent upon two or more responses for the TNBC cohort, and three or more for the HGSOC cohort, observed in the first stage. The planned sample size was rounded up to 30 per cohort to allow for an anticipated dropout rate of approximately 15–20%, ensuring that the required evaluable sample size for the design would be met. The null and alternative response rates were chosen based on clinically meaningful thresholds for response consistent with published response rates from trials evaluating PARPi in advanced breast and ovarian cancers with gBRCA1/2 mutations.

### Statistical analyses

The OTRR rate and 95% CI was calculated for each cohort using the normal approximation to the binomial. Duration of best response was defined as the time in months from the first scan indicating a response until they progressed or ended treatment, whichever came first. Progression-free survival (PFS) was defined as the time from registration to progression or death due to any cause, and participants who had not progressed or died were censored at the last known progression-free date. PFS included all participants enrolled. Analyses were performed in SAS version 9.4 (Cary, USA) and R version 4.1.3 [52].

### Correlative studies

Additional retrospective correlative studies were undertaken on all remaining tissue samples from the pre-screening program, on additional consented optional samples or samples from previous or subsequent biopsies retrieved from diagnostic pathology laboratories. Twenty such samples from 13 enrolled subjects were used to further investigate the effect of the proportion (quantitation) and extent (homogeneity) of promoter methylation, the impact of adjusting the methylation scores by tumour purity (where possible using the *TP53* PV VAF) and gene copy number (CN) and to identify potential mechanisms of PARPi resistance. The FFPE samples and matching germline DNA were examined by WES and quantitative NGS methylation assay as outlined in Supplementary Methods. Given the association between *gBRCA1* and me*BRCA1* status and TILs [20, 22], multiplex immunohistochemistry for T cell (CD4, CD8) and tumour cell markers (TNF2R, 7D3) were undertaken on the FFPE samples, as outlined in the Supplementary Methods.

## Results

### Subjects

**Fig. 1:**
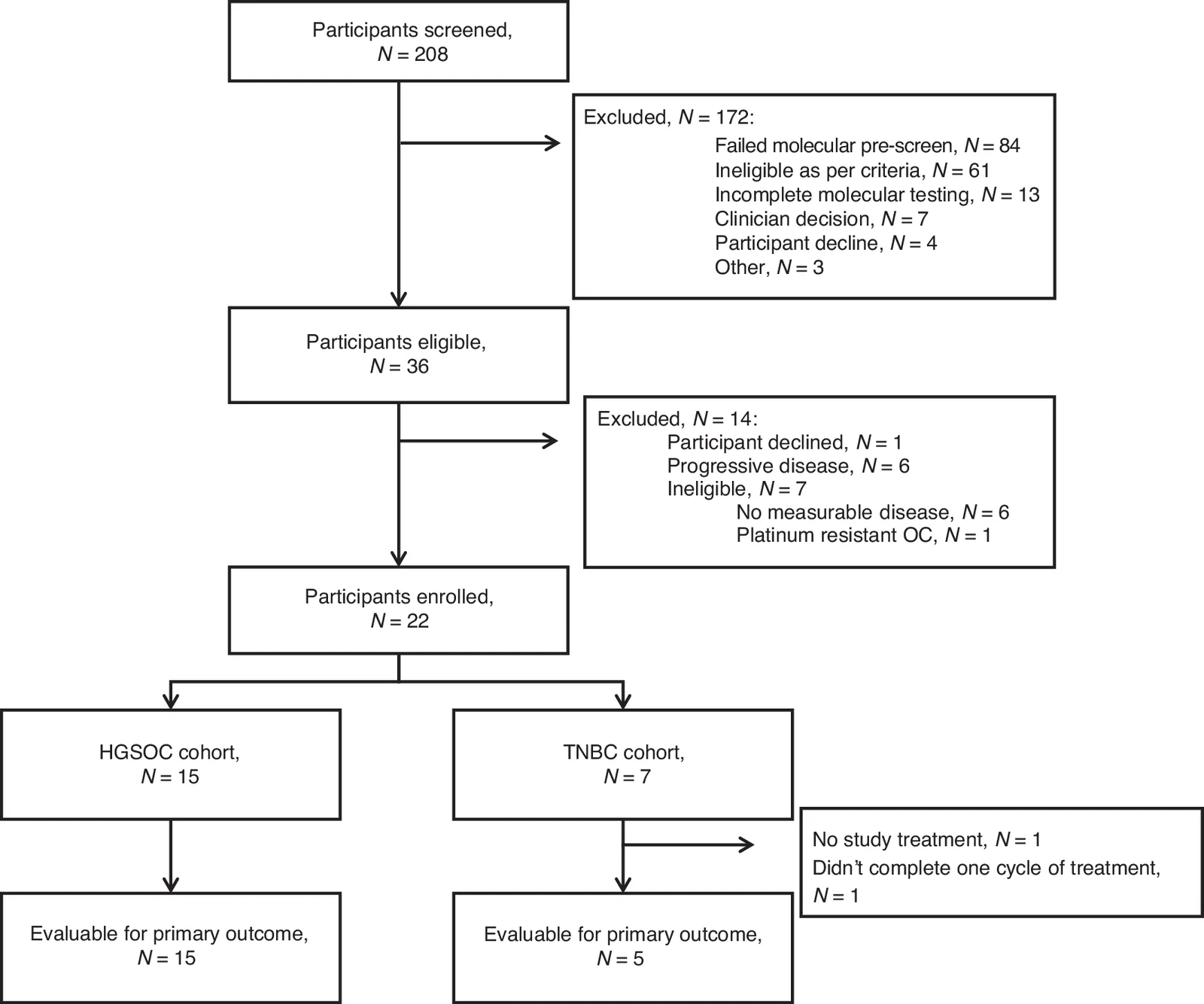
Participant flow diagram. The diagram illustrates participant disposition during pre-screening and study stages.

A total of 22 participants (15 HGSOC, 7 TNBC) were enrolled from 12 Australian centres between Sep 2018 and Mar 2022 (Fig. 1). Following data cut-off in Mar 2022 and study closure, five of 15 participants continued to receive olaparib via compassionate access (AstraZeneca), all from the HGSOC cohort. Of 208 pre-screened, 36 had cancers with eligible molecular characteristics. Of these, 22 enrolled.

Baseline characteristics of enrolled participants are outlined in Table 1. The median age was 61 years (range 56 – 72) and 56 years (range 55 – 64) for the HGSOC and TNBC cohorts respectively. All participants in the HGSOC cohort had received the required platinum-based chemotherapy prior to enrolment and for the TNBC cohort, 17% received prior platinum-based therapy and 83% received taxane-based chemotherapy (Table 1). In the HGSOC cohort, 73% (11/15) of participants had received only one prior line of chemotherapy. More than one prior line of platinum-based chemotherapy was allowed for participants with HGSOC containing germline mutations in non-BRCA genes. In the TNBC cohort, however, 50% had received two prior lines and 50% received three prior lines of treatment. All participants in the HGSOC cohort received study treatment, defined as at least one cycle of therapy, compared to 86% (6/7) in TNBC; and 83% of the HGSOC cohort received all planned treatment vs 67% of the TNBC cohort. Two participants in the TNBC cohort were non-evaluable for response; one did not commence treatment (#22) and the other (#07) did not complete the first cycle nor have a RECIST assessment post-baseline imaging. The median duration of treatment in the HGSOC and TNBC cohorts was 6.5 months and 2.6 months, respectively. The majority of participants ceased trial treatment due to disease progression (53% HGSOC vs 71% TNBC), with two participants ceasing treatment due to toxicity in the HGSOC cohort (13%; 2/15) compared with none in the TNBC cohort (0/7). The minimum number of responses was achieved in stage 1 for the HGSOC cohort to expand; however, the trial was closed.

**Table 1:** Baseline characteristics including genomic events.

Characteristic	HGSOC, *N* = 15^a^		TNBC, *N* = 7^a^	
Age (years)	61 (56, 72)		56 (55, 64)	
BMI (kg/m^2^)^b^	28.9 (25.2, 30.4)		29.1 (28.4, 33.0)	
ECOG status				
*0*	7 (47%)		0 (0%)	
*1*	8 (53%)		7 (100%)	
**Initial diagnosis**				
Years since initial diagnosis	3.91 (2.03, 5.78)		1.31 (1.00, 2.28)	
Primary site				
*Breast*	0 (0%)		7 (100%)	
*Ovary*	15 (100%)		0 (0%)	
Primary histological type				
*High-Grade Serous Carcinoma*	15 (100%)		0 (0%)	
*Infiltrating ductal carcinoma*	0 (0%)		7 (100%)	
**Metastatic recurrence**				
Months since diagnosis of stage IV/ recurrent disease	4 (1, 9)		5 (4, 10)	
Visceral	3 (20%)		4 (57%)	
Peritoneal	9 (60%)		0 (0%)	
Bone	0 (0%)		2 (29%)	
Breast	0 (0%)		2 (29%)	
Lymph nodes	7 (47%)		5 (71%)	
Other disease site				
*No*	14 (93%)		6 (86%)	
*Yes*	1 (6.7%)		1 (14%)	
Lines of treatment (total)^b^				
*1*	11 (73%)		0 (0%)	
*2*	2 (13%)		3 (50%)	
*3*	1 (6.7%)		3 (50%)	
*5*	1 (6.7%)		0 (0%)	
**Previous treatment received**^b,c^				
Taxane	14 (93%)		5 (83%)	
Platinum	15 (100%)		1 (17%)	
Anthracycline	3 (20%)		4 (67%)	
Alkylating agent	0 (0%)		4 (67%)	
Antimetabolite	2 (13%)		5 (83%)	
Immunotherapy	2 (13%)		0 (0%)	
VEGF inhibitor	4 (27%)		0 (0%)	
Hormone therapy	1 (6.7%)		0 (0%)	
Nontaxane microtubule inhibitor	0 (0%)		2 (33%)	
Tyrosine kinase inhibitor	1 (6.7%)		0 (0%)	
Vinca alkaloid	0 (0%)		2 (33%)	
Topoisomerase inhibitor	1 (6.7%)		0 (0%)	
**HR Deficiency (reviewed)**				
Methylation detected	8 (53%)		5 (71%)	
Mutation	5 (33%)		2 (29%)	
Signatures	2 (13%)		0 (0%)	
Methylation detected				
*BRCA1*	8		4	
*RAD51C*	0		1	
Mutation				
*gBRIP1*	1		0	
*gPALB2*	1		0	
*gRAD51C*	2		1	
*sBRCA1*	1		1	
Signatures				
HRD score	1		0	
Signature 3	1		0	
Methylation at screening (adjusted for tumour purity)	[*n* = 9] 63 (0, 81)		[*n* = 5] 28 (10, 89)	
Methylation at screening (adjusted for tumour purity) >70%	3 (33%)		1 (20%)	
Retrospective methylation (adjusted for tumour purity)	[*n* = 7] 89 (18, 100)		[*n* = 2] 60 (46, 74)	
Retrospective methylation (adjusted for tumour purity) >70%	6 (86%)		1 (50%)	

^a^Median (IQR); *n* (%).

^b^Multiple treatments may be used in each participant.

^c^One participant in the TNBC cohort had missing data.

### Safety

Overall, adverse events were consistent with published experience with olaparib in similar populations and are summarised in Table 2, with Grade 3 or higher AEs in Suppl. Table 1. AEs resulted in dose modification in three participants and cessation of treatment of two participants, all from the HGSOC cohort. No dose modifications or early cessation of treatment occurred for TNBC.

**Table 2:** Adverse events.

Adverse event*	Any AE grade		Grade 3 +	
	HGSOC, *N* = 15^a^	TNBC, *N* = 6^a^	HGSOC, *N* = 5^a^	TNBC, *N* = 3^a^
Nausea	10 (67%)	4 (67%)		
Fatigue	12 (80%)	2 (33%)		
Cough	4 (27%)	2 (33%)		
Vomiting	4 (27%)	2 (33%)		
Dysgeusia	5 (33%)	1 (17%)		
Abdominal pain	5 (33%)	1 (17%)	1 (20%)	0 (0%)
Anorexia	4 (27%)	2 (33%)		
Dyspnea	2 (13%)	2 (33%)	0 (0%)	1 (33%)
Back pain	4 (27%)	0 (0%)		
Dizziness	3 (20%)	0 (0%)		
Anemia	3 (20%)	0 (0%)	1 (20%)	0 (0%)
Constipation	1 (6.7%)	2 (33%)		
Rash maculo-papular	2 (13%)	1 (17%)		
Dry mouth	2 (13%)	1 (17%)		
Mucositis oral	2 (13%)	1 (17%)		
Anxiety	2 (13%)	1 (17%)		
Dyspepsia	2 (13%)	1 (17%)		
Small intestinal obstruction	2 (13%)	0 (0%)	2 (40%)	0 (0%)
Lung infection	1 (6.7%)	1 (17%)	1 (20%)	1 (33%)
Pneumonitis	1 (6.7%)	0 (0%)	1 (20%)	0 (0%)
Colonic obstruction	1 (6.7%)	0 (0%)	1 (20%)	0 (0%)
Thromboembolic event	0 (0%)	1 (17%)	0 (0%)	1 (33%)
Vasovagal reaction	1 (6.7%)	0 (0%)	1 (20%)	0 (0%)
Urinary tract infection	1 (6.7%)	0 (0%)	1 (20%)	0 (0%)
Heart failure	0 (0%)	1 (17%)	0 (0%)	1 (33%)

Summary of adverse events by tumour type (treated patients).

^a^n (%) * Events in at least 3 patients, or any grade 3 event has been reported.

### Genomic selection criteria analysis

Tumour cell content on all enrolled participants was estimated by a pathologist to be '>40% in all cases (range 40–98%, median = 70%). *BRCA1* and *RAD51C* promoter methylation > 4% was detected by MS-HRM in cancers of 53% (8/15) of participants in the HGSOC cohort (all me*BRCA1* - # 02, 05, 08, 09, 10, 12, 16, 21) and 71% (5/7) participants in the TNBC cohort (*n* = 4 me*BRCA1*, - # 01, 07, 18, 20 and *n* = 1 me*RAD51C -* # 13). Both cohorts included cases with heterozygous germline PVs (VAF 45–53%) in non-*BRCA* HR genes: *RAD51C* (*n* = 3 - #04 c.394dupA, p.(Thr132AsnfsTer23); #17 c.577 C > T, p.(Arg193Ter)); #19 c.904+5 G > T), *BRIP1* (#15; c2392C>T, p.(Arg798Ter)) and *PALB2 (#*14 c.2325dup, p.(Phe776IlefsTer26)) mutations. Two subjects had somatic *BRCA1* PVs: #06 (c.2680_2695dup. p.(Val899Glufs*9) sVAF = 40%, gVAF=0%) and #22 (3 Mb deletion spanning g.40811520-43922751, confirmed by WES). The cause of HRD was unclear in two HGSOC participants, one with mutational signature 3 (#03) and one with HRD-mean score of 20.67 (scores > 17.77 predicted to be deficient) (#11), as both were subsequently shown to be *BRCA1* and *RAD51C* unmethylated and wild-type for all HR genes. (Table 1).

### Primary outcome: best response by 6 months

**Fig. 2:**
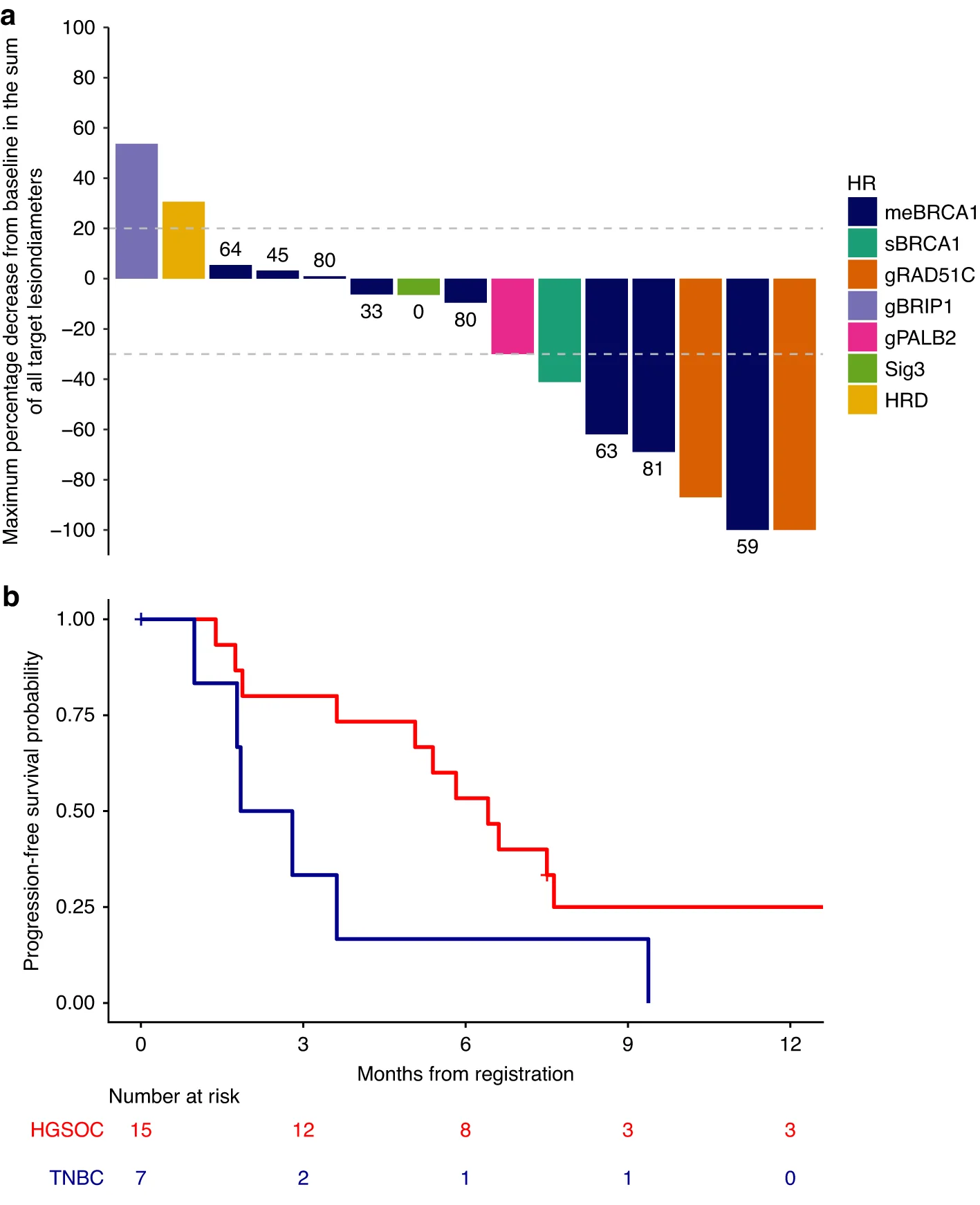
Responses according to percentage change in target lesions and progression free survival. **a** Waterfall plot for HGSOC cohort, coloured by HR event (*n* = 15). At baseline, the size of each target lesion (diameter in mm) was measured and summed. At each study visit, the sum of all target lesions was recalculated, and the percentage change from baseline was calculated. The largest decrease from baseline observed during followup is shown for each patient. Numbers indicate the methylation percentage (screening method) for each participant. **b** Kaplan-Meier for progression free survival by cohort (*n* = 22).

In the HGSOC cohort, the OTRR by 6 months was achieved in 6/15 participants in the HGSOC cohort (40%, 95% CI: 16–68%), including five with PR and one with CR (Suppl. Table 2, Fig. 2A). None of the seven participants in the TNBC cohort achieved OTRR at 6 months. A best response of stable disease (SD) by 6 months was achieved in 47% of participants (7/15) in the HGSOC cohort and 43% (3/7) in the TNBC cohort. Best response of disease progression by 6 months was reported for 13% (2/15) and 29% (2/7) participants in HGSOC and TNBC cohorts, respectively. Central and site assessments of response agreed after queries were resolved.

### Secondary outcomes

In the HGSOC cohort, the Kaplan-Meier estimated 6-month PFS was 53% (95% CI: 33–86%), and the 12-month PFS rate was 25% (95% CI: 10–62%). In the TNBC cohort, the Kaplan-Meier estimated 6-month PFS was 17% (95% CI: 3–100%), and the Kaplan-Meier estimated 12-month PFS was non-evaluable (NE). In total, 73% (11/15) and 86% (6/7) participants progressed in the HGSOC and TNBC cohorts, respectively. Median PFS in the HGSOC cohort was 6.4 months (95% CI: 5.1-NE) and 2.3 months (95% CI: 1.8-NE) in the TNBC cohort (Fig. 2b).

**Fig. 3:**
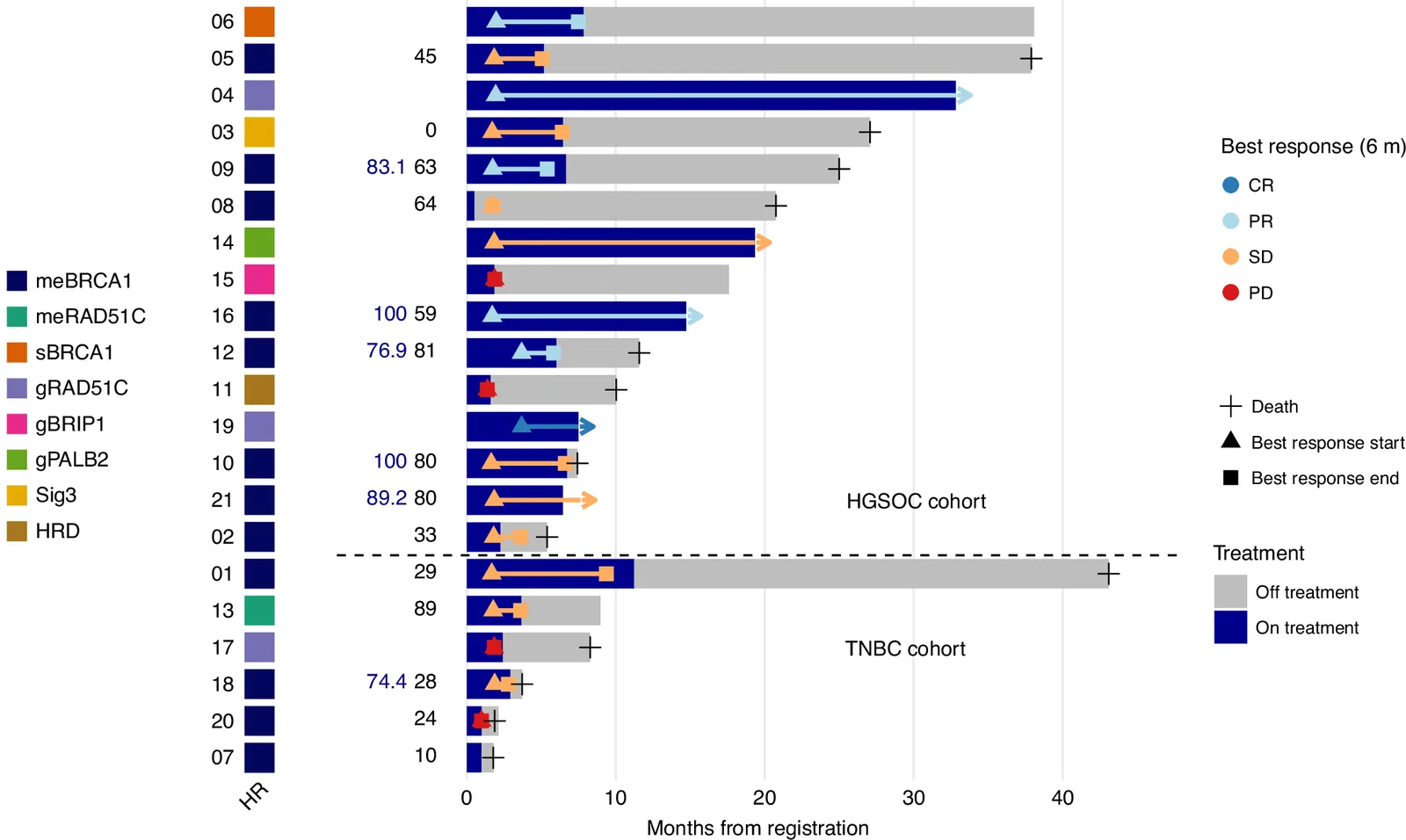
Swimmer plot of both cohorts, with each patient represented with bars indicating months on/off treatment (*n* = 21). The length of each patient’s best response by 6 months is shown on the bars, with arrows indicating those still responding at trial end. Black numbers indicate the proportion methylated (screening) and navy numbers indicate the retrospective proportion methylated. HR deficiency is shown on the far left.

In the post hoc analysis of the HGSOC cohort, median duration of best response was 3.8 months for CR (*n* = 1), 6 months for PR (*n* = 5), and 4.8 months for SD (*n* = 7). In the TNBC cohort, median duration was 1.8 months for SD (*n* = 3). One participant from each cohort experienced SD for longer than 6 months (Fig. 3).

In the HGSOC cohort, the OTRR rate of cases with detectable me*BRCA1* was 38% (3/8) (95% CI: 9% - 76%) versus 43% (4/7) (95% CI: 10–82%) for cases with HRD due to other causes (Suppl. Table 2). In the HGSOC cohort, the Kaplan-Meier estimated 6-month PFS was 38% in those with detectable me*BRCA1* vs 71% for cases with HRD due to other causes. In the TNBC cohort, the Kaplan-Meier estimated 6-month PFS was 20% in those with detectable me*BRCA1* or me*RAD51C* (*n* = 5) vs not estimable in the two cases with HRD due to other causes.

Of the HGSOC participants, 14/15 were evaluable for CA-125 response assessment, with a baseline sample at least twice the upper limit of normal. Of these, 60% (9/14) had CA-125 response. No association between lines of prior therapy and GCIG CA125 or RECIST 1.1 response was found in this small cohort (Suppl. Table 3).

### Tertiary outcomes: methylation

The enrolment criteria for detectable methylation using the MS-HRM pre-screening assay resulted in a heterogenous group of 13 participants with HRD due to promoter methylation of *BRCA1* or *RAD51C* (mutation-negative). When re-tested with the confirmatory quantitative Oncoreveal^TM^ *BRCA1* and *RAD51C* methylation test, and pathology review which determined tumour cellularity, the adjusted methylation levels ranged from 10 to 89% (median = 50%).

Best response in the 12 me*BRCA1* participants was predominantly SD at 62% (5/8) within the HGSOC group and 50% (2/4) in the TNBC cohort. A best response of PR was achieved in 38% (3/8) of HGSOC, but none of the TNBC, subjects (Suppl. Table 2). When the proportion of methylation was quantified and adjusted for tumour cellularity, there was heterogeneous response within the me*BRCA1* methylated HGSOC cohort, with methylation levels of 59% (#16), 63% (#09) and 81% (#12) in the responders and 33% (#02), 45% (#05), 64% (#08), 80% (#10 and #21) in the non-responders (Fig. 2). Notably, one subject (#16) with 59% methylation, had a durable partial response (Fig. 3). Best response for *RAD51C* methylation was only assessable in the one patient, with TNBC (#13) with 89% methylation, who achieved a best response of SD. Notably, this subject’s tumour was subsequently shown to be HR proficient with our WES HRD assay (Suppl. Table 5).

In an attempt to understand the impact of adjusting methylation proportion by different methods, we retrospectively assessed methylation levels adjusted by tumour purity and gene CN, determined by WES, and the extent of methylation (number of methylated promoter CpG alleles), determined by quantitative NGS methylation assay (described in Supplementary methods), on matched samples (same block and timepoint) from six participants with me*BRCA1* or me*RAD51C* (#09, #10, #12, #16, #18, #21). In all six cases, the WES-adjusted methylation was > 70% with three subjects increasing from < 70% initially (#09, #16, #18) with three achieving a PR (#09, #12, #16), and three SD (#10, #18, #21) (Fig. 3, Suppl. Table 4). Time on treatment for these subjects varied, with 4 stopping treatment for disease progression; of these, the time to progression was 3 months (#18, TNBC) to approximately 6 months (#9, #10, #12 HGSOC). Two subjects continued to respond at study closure, after 8 (#21) and 15 months (#16) and were transitioned to compassionate access.

Analysis of the extent of methylation showed that the *BRCA1* promoter region is homogenously methylated or unmethylated at all nine CpG sites assessed (Suppl. Fig. 1A), indicating that variation in the proportion (% of methylation) in methylated cases results from a variable admixture of methylated tumour cells and unmethylated non-tumour cells, arising from variable tumour cellularity. When adjusted for bioinformatically-calculated tumour purity (WES analysis) (Suppl. Table 4), seven of the nine *BRCA1* methylated cases were > 70%, compared to three of nine cases with the pathologist-determined tumour cellularity estimate, in the samples re-tested by the quantitative NGS methylation assay for which appropriate material was available. These observations highlight the importance of incorporating accurate tumour cellularity and purity measurements in methylation testing to predict potential responders.

### Tertiary outcomes: non-g*BRCA* HR mutations

Of the two HGSOC subjects with g*RAD51C* PV, both had deep and durable responses; one (#19) experienced a CR and another (#04) a PR, supporting previous pre-clinical and clinical reports [27] that *RAD51C* is a critical HR gene and biomarker for PARPi response (Fig. 3) [33, 53]. The TNBC subject (#17) with g*RAD51C* PV experienced PD following two lines of prior treatment, indicating the likely emergence of resistance mutations [27] One HGSOC subject with a s*BRCA1* PV (#6) and the HGSOC subject with the g*PALB2* (#14) PV experienced PR and SD, respectively, consistent with previous reports [54]. The tumour from the HGSOC subject with a g*BRIP1* PV (#15) showed somatic loss of heterozygosity (LOH) (tumour VAF 84%) and experienced PD, consistent with previous reports that not all *BRIP1* PVs are a biomarker of PARPi sensitivity, even with biallelic loss [55]. The unmethylated *BRCA1* and *BRCA2* wild-type HGSOC subjects with a low HRD score (#11) or signature 3 (#3) experienced PR and SD, respectively. Notably, the patient with Signature 3 (#3) was subsequently shown to be HR proficient on our WES assay (Supplementary Table 5), questioning the veracity of the signature in this case.

### Tertiary outcomes: co-occurring mutations

**Fig. 4:**
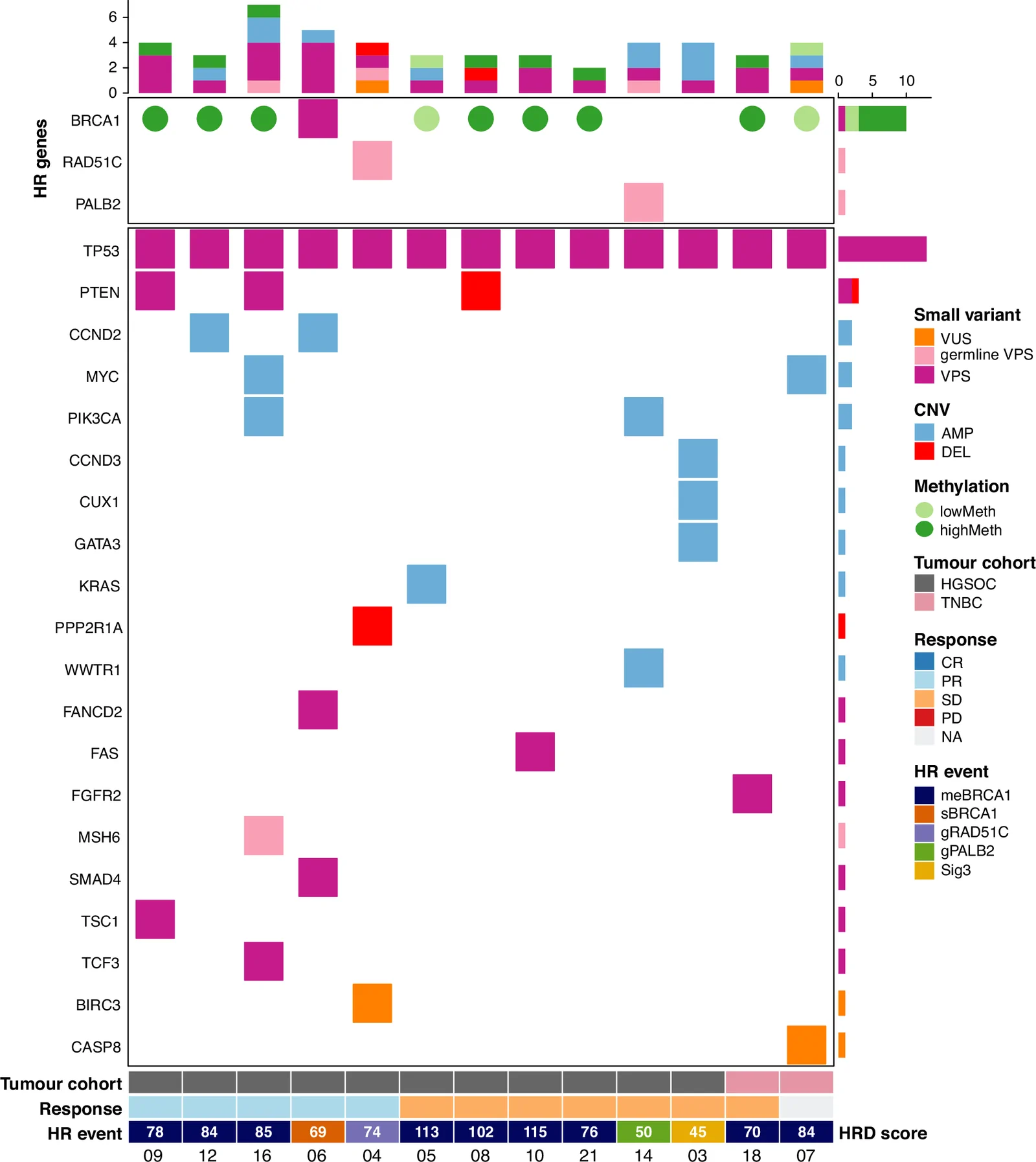
Molecular characterization of the EMBRACE cohort. WES was performed on matched tumour and germline DNA samples. Curated pathogenic variants (VPS), variants of uncertain significance (VUS), and copy number variants (CNV), either amplifications (AMP) or deletions (DEL) are shown by the squares. Results from retrospective targeted amplicon methylation analysis are indicated by the green circles (where high is ³70% WES tumour purity-adjusted methylation, and low is < 70%). Response = best response at 6 months.

For the 13 participants with tumour tissue available for retrospective correlative study, WES was performed to identify additional molecular aberrations that could impact on treatment response (Fig. 4). As expected for HGSOC and TNBC, all 13 cases harboured somatic *TP53* PVs. The next most frequent events were *PTEN* deletions and splice variants (three cases) and amplifications of *MYC* (two cases), *PIK3CA* (two cases) and *KRAS* (one case), all known to be associated with drug resistance in HGSOC, however, there was no obvious association of potential resistance mutations with response in this cohort. Although frequent in HGSOC, amplification of *CCNE1* was not observed in this platinum-sensitive cohort, consistent with previous studies reporting that *CCNE1* amplification occurs predominantly in platinum-refractory disease and is mutually exclusive of *BRCA1* PV [28].

### Tertiary outcomes: immune filtration

CD4 and CD8 tumour infiltrating lymphocytes (TILs) were assessed using OPAL multiplex immunohistochemistry (described in Suppl. Fig. 2) on 20 samples (16 HGSOC from 13 participants and four TNBC from four participants). High total TILs (combined CD4 and CD8 counts) (*p* = 0.022) and CD4 counts (*p* = 0.014) but not CD8 counts (*p* = not significant) were associated with a better clinical response (CR_PR v SD_PD) in HGSOC, consistent with a previous finding of higher TILs in non-BRCA HRD tumour compared to HR-competent tumours [22]. There was no association between CD4 or CD8 TILs with PFS (split at < 6 months compared to ≥ 6 months) or OS ( < 12 months compared with ≥ 12 months).

## Discussion

The EMBRACE trial demonstrated that activity of olaparib monotherapy in HGSOC with HRD other than gBRCAm was comparable to that seen in gBRCAm HGSOC, with OTRR of 40%. Notably, durable responses to olaparib were achieved in a subset of HGSOC subjects with high levels of *BRCA1* promoter methylation or *gRAD51C* mutations. We did not observe any objective responses in the TNBC cohort with best response achieved being SD in 43% of participants. This contrasts with recent case reports of patients with metastatic TNBC with *gBRCA1* mutations or homozygous *BRCA1* methylation having exquisite sensitivity to platinum as an initial therapy [37]. Notably, the 50% of TNBC participants who had received three prior lines of therapy had adjusted methylation of 10%, 29% and 28% suggesting that biallelic *BRCA1* or *RAD51C* methylation has been partially lost as a result of prior treatment.

Although the proportion women with HGSOC and TNBC with *BRCA1* promoter methylation is estimated to be 10–20% and 29–46% [13–15] respectively, it is currently unclear what proportion have biallelic (homozygous) vs monoallelic (heterozygous) methylation and therefore could potentially be sensitive or refractory to PARPi, respectively. Since *gBRCA1* and *meBRCA1* cases have equivalent prognosis and shared morphological features, it is not unreasonable to assume that epigenetic silencing of *BRCA1* is a tumour initiating event that causes HRD in such cases and that biallelic (homozygous) methylation persists until subsequent mutational or adaptive cellular events at other genes begin to drive the tumour and relieve its reliance on *BRCA1 or RAD51C* silencing.

The mechanisms, dynamics and prevalence of methylation loss, particularly in younger women and early-stage disease are poorly understood. What is clear, however, is that a currently unknown proportion of HGSOC and TNBC may be initiated by epigenetic silencing of either *BRCA1* or *RAD51C* and retain biallelic (homozygous) methylation into the metastatic disease setting and sometimes even through multiple lines of treatment. These women, with potentially unstable tumour promoter methylation, are not currently identified by any approved diagnostic tests including NGS panels, HRD genomic instability scores (GIS) or mutational signatures. Moreover, because HRD genomic scars are permanent, rather than contemporaneous, they may give a false impression of functional HRD status and likely PARPi responsiveness in patients with *BRCA1* or *RAD51C* promoter methylation whose tumours were initially HRD but regained HR proficiency due to later loss of epigenetic silencing.

The restriction on limiting the number of prior lines of platinum treatment, the requirement to pre-screen large numbers of patients to identify the rare subset that met the eligibility criteria, and the time taken to retrieve archival samples and to complete the serial methylation and HRD testing with confirmation of the absence of *gBRCA1* or *gBRCA2* PVs, all combined to create enrolment challenges and delays. These were further compounded by pausing the pre-screening program twice during COVID and the need to reestablish and revalidate the assay following withdrawal of the first diagnostic laboratory providing the testing. Accordingly, the trial was prematurely terminated with only 22 of the planned 60 patients enrolled. Consequently, the study was underpowered to address the main hypothesis. Conclusions regarding secondary and tertiary outcomes are similarly limited with wide confidence intervals reflecting the small sample size.

Nevertheless, important clinical signals were identified including the demonstration that a subset of HGSOC participants with high-level (presumably homozygous) *BRCA1* promoter methylation or *gRAD51C* PVs experienced either CR or long-term durable responses to olaparib as a monotherapy. Furthermore, the lack of response to olaparib in pretreated meTNBC is likely due to the dynamic nature of promoter methylation and its propensity to be lost under selective treatment pressure especially to platinum therapy. Our study highlights a potential difference in me*BRCA1* stability between HGSOC and TNBC, which is likely treatment-related and should be explored further.

Our observations have important implications for clinical practice guidelines, future research and clinical trials. Notwithstanding the challenges of pre-screening and identifying TNBC patients with homozygous me*BRCA1* or me*RAD51C* and then enrolling them in a PARPi trial early in their disease journey, ideally prior to platinum exposure, it is clear that there is a subset of patients who could potentially benefit from PARPi if they could be identified and treated early enough. Presently, the prevalence of homozygous me*BRCA1* and me*RAD51C* in the population of women with early breast or ovarian cancer is unknown. This study led to the development (by Xing Technologies) and commercialisation (by Pillar Biosciences) of a highly sensitive and robust quantitative *BRCA1* and *RAD51* methylation assay [42] that could be used to screen patients for next-generation PARPi trials, particularly neoadjuvant trials in early stage TNBC.

Additionally, our work in the EMBRACE, ARIEL 2 [29] and DORA [49] trials demonstrates that long-term durable PARPi responses can be achieved in patients who maintain homozygous promoter methylation despite selective treatment pressures. Understanding the mechanism of promoter demethylation and exploring ways to ways to prevent loss of methylation has the potential to improve the durability and overall survival benefits of PARPi in HGSOC and TNBC patients without *gBRCA* or HR gene PVs, whose cancers are potentially initiated by epigenetic silencing of either *BRCA1* or *RAD51C*.

## Conclusion

Olaparib demonstrated clinically relevant activity in HGSOC without gBRCAm. This is the first trial to demonstrate response to PARPi in prospectively selected HGSOC with HRD due to promoter methylation of *BRCA1* or *RAD51C*. Objective responses were seen in HGSOC with me*BRCA1*. Olaparib had limited activity in TNBC with two or three prior lines of therapy. Further research is needed into the effects of chemotherapy on the proportion and extent of promoter methylation of *BRCA1/RAD51C*, in order to improve selection of patients with HGSOC and TNBC for PARPi therapy.

### Translational relevance

This is the first trial to demonstrate response to PARPi in prospectively selected cases of HGSOC with HRD due to promoter methylation of *BRCA1*. Objective responses were seen in HGSOC with *BRCA1* promoter methylation or germline *RAD51C* pathogenic variants. Olaparib had limited activity in TNBC with either *BRCA1* or *RAD51C* promoter methylation, all of which had received prior exposure to two or three lines of therapy. Further research is needed into the effects of PARPi and platinum and other chemotherapy on *BRCA1/RAD51C* promoter stability and acquired demethylation in order to improve selection and ensure durable PARPi responses of patients with HGSOC and TNBC.

## Supplementary information

Supplementary Material - methods, tables & figures

## Data Availability

The data generated in this study are available upon request from the corresponding author.
