# Supplementary material for: Olaparib in HR-deficient, metastatic triple-negative breast and platinum-sensitive relapsed ovarian cancers without germline mutations in BRCA1/2: phase 2 EMBRACE trial
Source: Br J Cancer. 2026 Jul 8;135(7):1152–63. doi: 10.1038/s41416-026-03535-6 (PMC13578435; doi:10.1038/s41416-026-03535-6)
Supplement: Supplementary file 1 [file 41416_2026_3535_MOESM1_ESM.pdf]

SUPPLEMENTARY METHODS

Retrospective molecular analysis

To confirm and cross-validate the tumour-specific methylation percentage, targeted bisulfite amplicon NGS methylation testing of *BRCA1* and *RAD51C* promoter regions was performed where possible, in addition to the primary methylation screening (methylation-sensitive high-resolution melting PCR and Pillar Biosciences *BRCA1 & RAD51C* Methylation test). DNA was extracted using QIAamp DNA FFPE tissue kit (Qiagen) from additional FFPE tumour tissue sections from the same or alternative FFPE tumour blocks as those used for trial screening, where tissue was available. Initial tumour cell purity was estimated by pathologist review, and only samples with >10% purity estimations were tested.

Bisulfite DNA conversion was performed using EZ DNA Methylation-Lightning kit (Zymo Research). Targeted bisulfite amplicon RAD51C promoter methylation NGS was performed as previously described(1). *BRCA1* promoter bisulfite amplicon NGS was performed using the same approach with the following primers:

Forward primer (5'-3'), Illumina sequencing adaptors as bolded text:
TCGTCGGCAGCGTCAGATGTGTATAAGAGACAGTtgTtgTttagcggtagTTTTttggt;

Reverse primer (5'-3'), Illumina sequencing adaptors as bolded text:
GTCTCGTGGGCTCGGAGATGTGTATAAGAGACAGaAcctAtcccccgtccaAAaa

Sequencing was performed using MiSeq platform (Illumina) 2x150bp with mean read depth per sample of >7000x achieved for *BRCA1* and >6000x for *RAD51C* across all samples. Methylation analysis was performed using *MethAmplicons2* (https://github.com/molonc-lab/MethAmplicons2), using a 0.01 read frequency cut-off (1). All samples were found to have an acceptably high bisulfite conversion efficiency (>98%). "Fully methylated epialleles" for each gene were defined as follows: epialleles with 8/9 CpG sites methylated for the *BRCA1* promoter, and 7/13 CpG sites methylated for the *RAD51C* promoter, as previously described(1). Adjusted tumour methylation was calculated using previously published formulas, which incorporate tumour purity and gene copy number estimates (1,2). Final tumour purity, *BRCA1* and *RAD51C* gene copy number and tumour co-mutations were estimated using whole-exome sequencing (WES).

DNA was extracted from FFPE tumour (as above) and germline DNA from blood using the QIAamp DNA mini kit. Library preparation for WES was performed with Twist Exome v2 (Twist Biosciences, USA) with IDT xGen cfDNA & FFPE library prep (Integrated DNA Technologies, Coralville, USA) with Unique Molecule Identifiers and using mechanical shearing. Libraries were sequenced to a target depth of 20Gbp with 150PE reads (200x) on the Illumina Novaseq X plus platform. Library preparation and sequencing was performed by the Australian Genome Research Facility (Melbourne, AUS).

Tumour samples and matched germline DNA were analysed using a bionix pipeline (https://github.com/PapenfussLab/bionix) (3) as follows. Reads with duplicate UMIs were removed using dedumi (0.1) (https://github.com/PapenfussLab/dedumi/

) and aligned to GRCh38p31 (4) with StrobeAlign (0.11.0) (5). Octopus (v0.7.0) (6) was used to call and phase SNVs and indels with subsequent annotation against Ensembl (v110) (7) within regions ± 100 bp of exon boundaries. Variants were annotated with dbNSFP (v4.2a)(8) using SnpEff and SnpSift (v4.3t) (9). Copy number variants were called using FACETS (0.6.1) (10). HRD status was called following Marquard et al. (11) using their published code updated to GRCh38 and applied to copy number calls from FACETS. Scores >55 were defined as HRD based on calibration to known samples.

Variant Curation

Germline variant lists collated from processed WES data were ordered by variant allele frequency (AF) plus the presence of the variant in the Familial Cancer Centre (FCC) database (i.e. highest AF with an FCC entry first). Variants in the blood with allele frequencies lower than 50% with a buffer of +/- 10-15%(i.e. below the expected AF for heterozygosity) were not investigated further. Loss of heterozygosity of a germline variant in the tumour was determined by change in AF in the tumour sample compared to the blood.

Collated somatic variants were ordered by haplotype frequency (HF) plus the presence of the variant in the COSMIC database (i.e. highest HF with a COSMIC entry first). Variants were investigated from the highest AF (usually a percentage equivalent to the purity of tumour) down to an AF of ~10%.

The likelihood of pathogenicity for each variant of interest was determined using information from the following databases: OncoKB (https://www.oncokb.org/); Clinvar (https://www.ncbi.nlm.nih.gov/clinvar/) and the GENIE Cohort v15.1-public database (https://genie.cbioportal.org/). Evidence for the pathogenicity of copy number variants (CNVs) was determined using information from OncoKB. Only transcript variants or CNVs with clear evidence of pathogenicity were reported for each case. Those with conflicting evidence of pathogenicity were classed as variants of potential significance.

Multiplex Imaging and analysis

OPAL™ 6-plex multiplex immunohistochemistry was performed on all available FFPE tumour samples. Each 4-5um section was probed with CD8 (4B11, Invitrogen), CD4 (EPR6855/4B12, Abcam) and a tumour marker (TNFR2; 7D3, ThermoFisher Scientific) along with three additional antibodies (not reported in this study) using the recommended protocol with optimized staining sequence and DAPI as the nuclear stain. Stained tissues were imaged using the Vectra Polaris™ (Akoya Biosciences) and broad regions of interest were pre-processed using PhenoChart™ (Akoya Biosciences) whole slide viewer and inForm tissue analysis software (Akoya Biosciences). Targeted regions of interest were further processed in QuPath(12) using StarDist (13) for nuclear/cell detection and a set of custom trained classifiers to identify tumour areas and define individual cell annotations. Due to tumour/sample variability, tumours were processed with one of four different tumour classifiers. Tumour and adjacent stroma classification was informed by comparison with previous H&E sections and selection discussed with a pathologist. Any images with insufficient tumour or poor global staining were removed from further analysis. The tumour area included the interacting adjacent stromal cells within 100mm of the tumour. All cell annotations were defined using the same set of object classifiers defined using a machine learning approach built into QuPath. Imaging data was processed using R (14) and Tidyverse package version 4.4.0(15) while the results were visualised using ComplexHeatmap(16). A Wilcoxon rank sum test was used to assess differences in detected tumour infiltrating lymphocytes (TILs) between groups.

Supplementary TABLES and FIGURES

Supplementary Table 1

Supplementary Table 1: Worst grade AE reported by tumour type

**Characteristic**	**HGSOC**, N = 15[1]	**TNBC**, N = 6[1]
Maximum grade reported		
1	2 (13%)	1 (17%)
2	8 (53%)	2 (33%)
3	5 (33%)	3 (50%)

[1]n (%)

Supplementary Table 2: response rate

Best response (confirmed) by tumour type, and methylation status

	Overall		By methylation status			
			HGSOC		TNBC	
**Characteristic**	**HGSOC**, N = 15[1]	**TNBC**, N = 7[1]	**Other**, N = 7[1]	**Methylated**, N = 8[1]	**Other**, N = 2[1]	**Methylated**, N = 5[1]
Best response by 6 months						
NE - Not Evaluable	0 (0%)	2 (29%)	0 (0%)	0 (0%)	1 (50%)	1 (20%)
CR - Complete Response	1 (6.7%)	0 (0%)	1 (14%)	0 (0%)	0 (0%)	0 (0%)
PR - Partial Response	5 (33%)	0 (0%)	2 (29%)	3 (38%)	0 (0%)	0 (0%)
SD - Stable Disease	7 (47%)	3 (43%)	2 (29%)	5 (62%)	0 (0%)	3 (60%)
PD - Progressive Disease	2 (13%)	2 (29%)	2 (29%)	0 (0%)	1 (50%)	1 (20%)
Overall response rate (95% CI)	6/15; 40% (16%, 68%)	0/5; 0% (0.00%, 52%)	3/7; 43% (9.9%, 82%)	3/8; 38% (8.5%, 76%)	0/1; 0% (0.00%, 98%)	0/4; 0% (0.00%, 60%)

[1]n (%)

Supplementary Table 3

Table S3: Percentage of patients with each line of therapy for a given best response by tumour type, for all participants and methylated only

	All participants						Methylated only				
**Tumour type**	**HGSOC**				**TNBC**		**HGSOC**			**TNBC**	
**Lines of treatment**	**1 line**	**2 lines**	**3 lines**	**5 lines**	**2 lines**	**3 lines**	**1 line**	**2 lines**	**5 lines**	**2 lines**	**3 lines**
N	11	2	1	1	3	3	6	1	1	2	3
Best response by 6 months											
NE - Not Evaluable						1 (100%)					1 (100%)
CR - Complete Response	1 (100%)										
PR - Partial Response	4 (80%)	1 (20%)					2 (67%)	1 (33%)			
SD - Stable Disease	5 (71%)	1 (14%)		1 (14%)	1 (33%)	2 (67%)	4 (80%)		1 (20%)	1 (33%)	2 (67%)
PD - Progressive Disease	1 (50%)		1 (50%)		2 (100%)					1 (100%)	0 (0%)

[1]n (%) Percentages are row percentages for that cohort's tumour type; ie. In all HGSOC patients, 80% of those whom had PR had one line of treatment.

Supplementary Table 4

Methylation percentage according to adjustment method, and association with response in matched samples for participants with *meBRCA1* or *meRAD51C.*

Participant ID	Cohort	Best response by 6 months	Methylation (%)			
			Unadjusted (Pillar)	Adjusted - tumour purity (Pillar)	Unadjusted meNGS	Adjusted meNGS – tumour purity and gene copy number (WES)
09	HGSOC	PR	60	63	54	83.1
10	HGSOC	SD	40	80	43	100
12	HGSOC	PR	40	81	30	76.9
16	HGSOC	PR	50	59	74	100
18	TNBC	SD	20	28	29	74.4
20	TNBC	PD	10	24	-	-
21	HGSOC	SD	60	80	71	89.2

*= retrospective

Supplementary Table 5

HRD Status and HRD score from post enrolment WES assay. Scores >55 are HRD in this assay (Lee et al 2025). HRD score was determined for two samples from participant 6.

Participant ID	Cohort	HRD type	HRD score	Best Response
3	HGSOC	Sig3	45	SD
4	HGSOC	gRAD51C	74	PR
5	HGSOC	meBRCA1	113	SD
6	HGSOC	sBRCA1	69,73	PR
7	TNBC	meBRCA1	84	PD
8	HGSOC	meBRCA1	102	SD
9	HGSOC	meBRCA1	78	PR
10	HGSOC	meBRCA1	115	SD
12	HGSOC	meBRCA1	84	PR
14	HGSOC	meRAD51C	50	SD
16	HGSOC	meBRCA1	85	PR
18	TNBC	meBRCA1	70	SD
21	HGSOC	meBRCA1	76	SD

Supplementary Figure 1. **Allele specific analysis of *BRCA1* and *RAD51C* promoter methylation. (A)** *BRCA1* promoter methylation and (**B)** *RAD51C* promoter methylation was assessed by targeted sequencing of bisulfite converted DNA. CpG sites within the corresponding promoter are represented as circles on the lollipop plot. The degree of methylation at each CpG site is indicated by the colour, with fully methylated CpG sites in red and unmethylated sites in blue. No samples were found to have *RAD51C* methylation. **(C)** The unadjusted percentage of *BRCA1* promoter methylation (defined as % of epialleles/ amplicons with ^{3} 8 out of 9 CpG sites methylated) is plotted with the percentage tumour purity as determined by WES. **(D)** The adjusted tumour percentage *BRCA1* promoter methylation was calculated using tumour purity and *BRCA1* copy number estimated from WES, as previously described [Kondrashova 2018 and Nesic 2021]. Samples with ^{3}70% adjusted methylation (dotted line) were defined as “highly methylated” in this study. Two samples with methylation <70% (orange shading) display reduced, or “low” methylation.

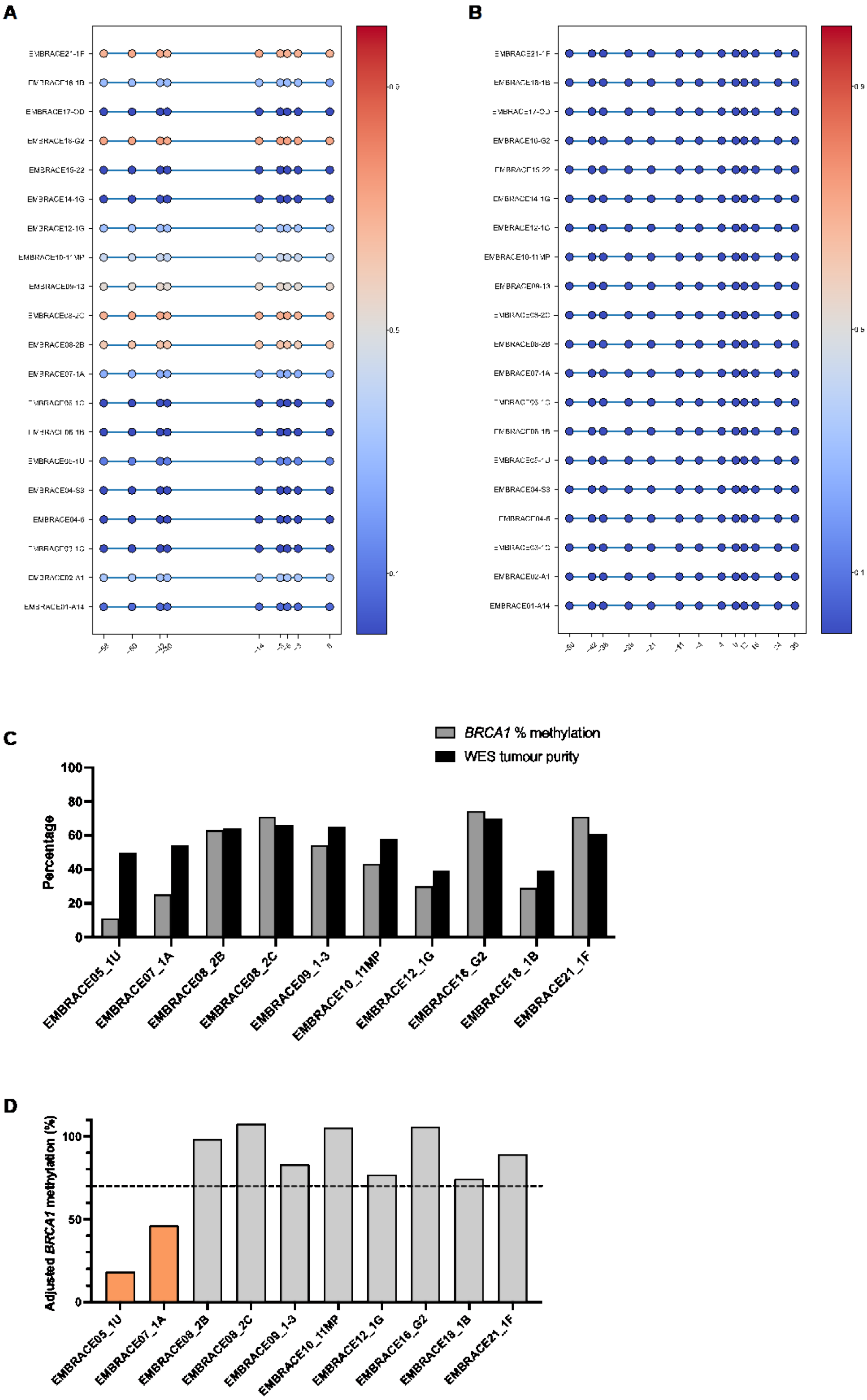
A
B
C
BRCA1 % methylation
WES tumour purity
Percentage
100
80
60
40
20
0
EMBRACE05_1U
EMBRACE07_1A
EMBRACE08_2B
EMBRACE08_2C
EMBRACE09_1-3
EMBRACE10_11MP
EMBRACE12_1G
EMBRACE16_G2
EMBRACE18_1B
EMBRACE21_1F
D
Adjusted BRCA1 methylation (%)
100
50
0
EMBRACE05_1U
EMBRACE07_1A
EMBRACE08_2B
EMBRACE08_2C
EMBRACE09_1-3
EMBRACE10_11MP
EMBRACE12_1G
EMBRACE16_G2
EMBRACE18_1B
EMBRACE21_1F

Supplementary Figure 2

Immune tumour landscape of the EMBRACE cohort

Evaluation of CD4 and CD8 T cell infiltration in archival tumours using OPAL multiplex IHC imaging. (A); CD4 and CD8 T cell counts as a proportion of all cells in the tumour and adjacent stroma (adjacent stroma extends 100um from tumour-stroma boundary) ordered by clinical response and combined T cell count. (B) Representative OPAL images of a tumour from participants who achieved PR (06) or SD (05) on trial. Smaller panels are the individual channels with the larger combined images underneath. Blue; DAPI, cyan; CD4+, yellow; CD8+ and pink; TNFR2 used as tumour marker. Size bar; 250µm (C) Combined CD4 and CD8 T cell counts comparing tumours from participants grouped by CR/PR v SD/PD (p = 0.022, Wilcoxon rank sum test)

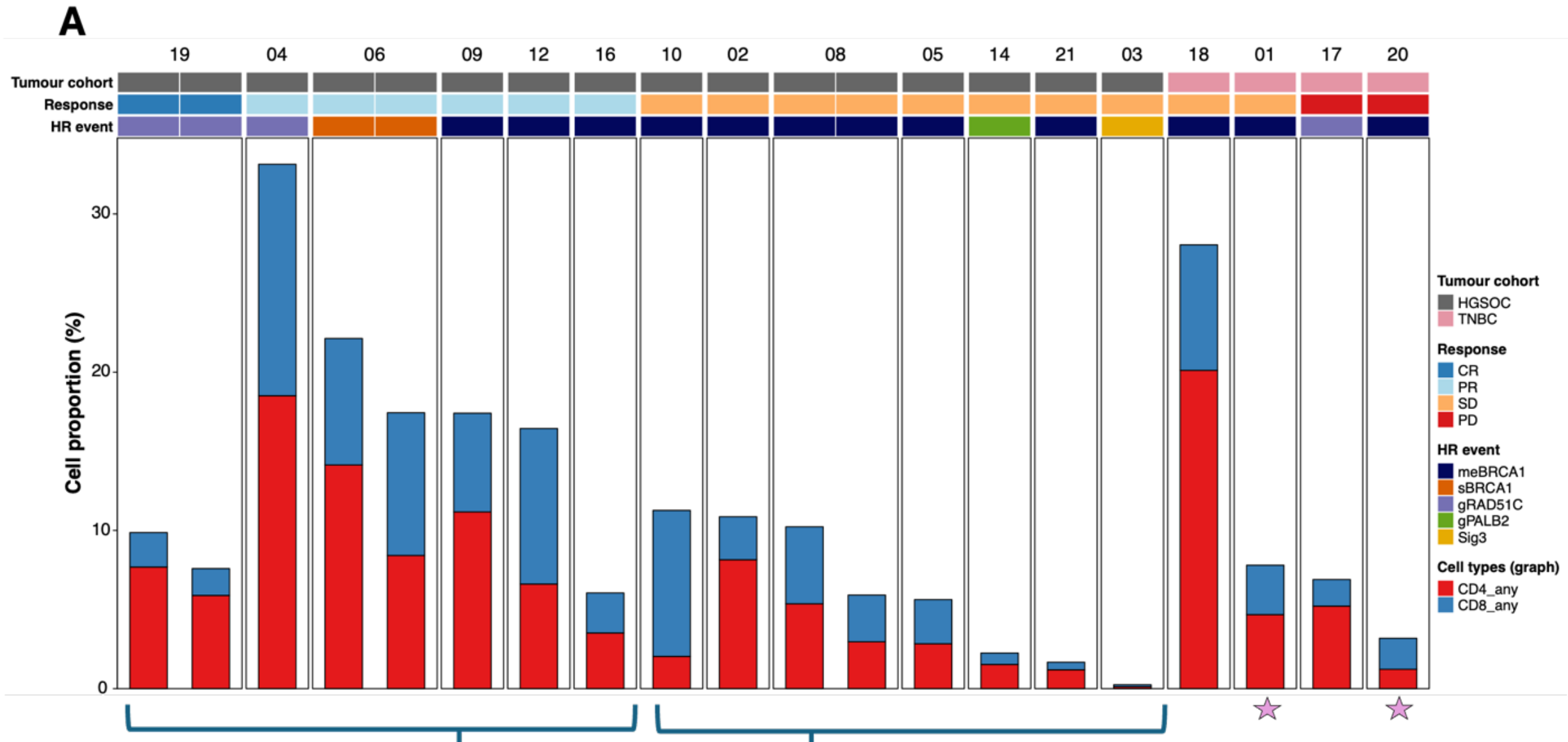

Total TILS*	p=0.022
CD4*	p=0.014
CD8	p=0.138

☆ <2500 tumour cells

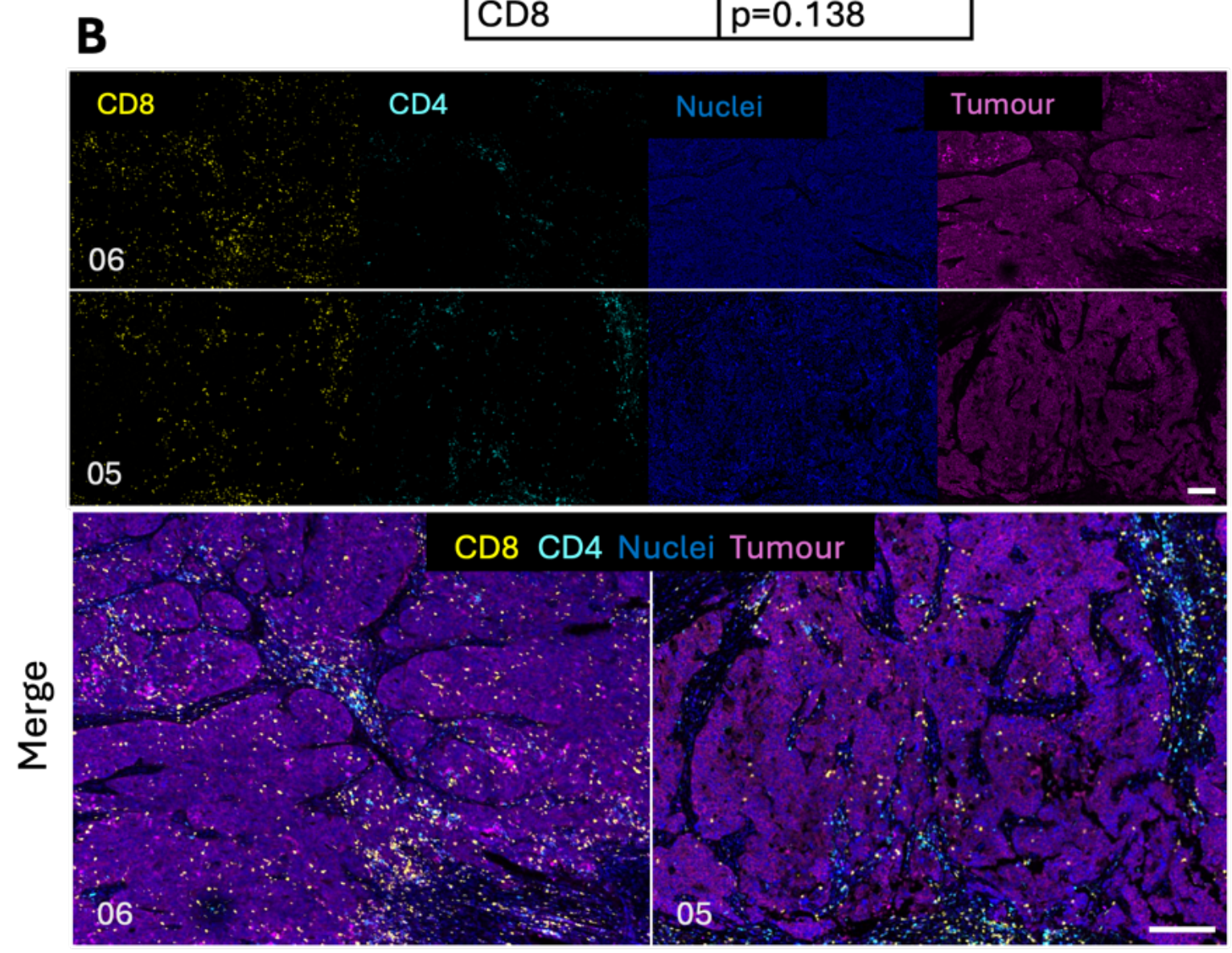

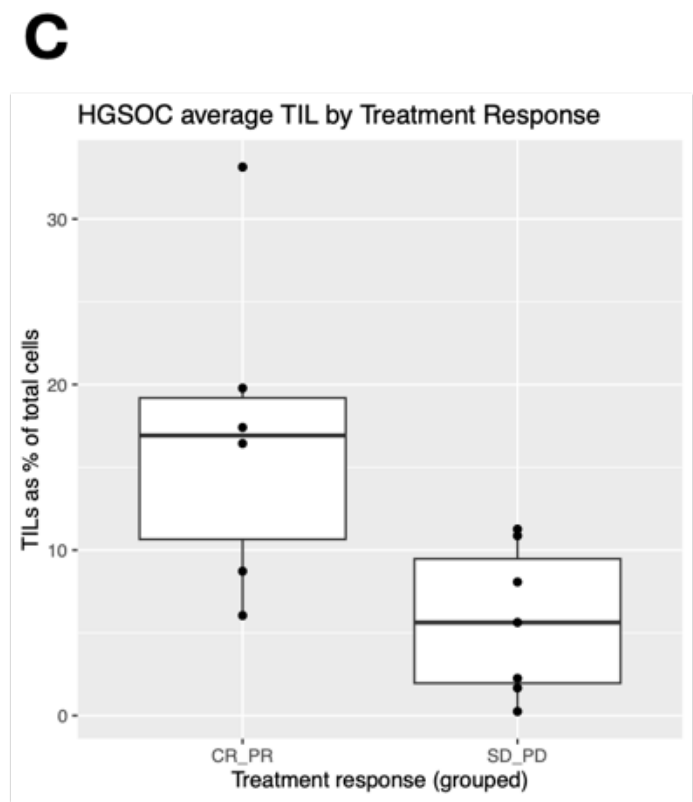

REFERENCES

1. Nesic K, Kondrashova O, Hurley RM, McGehee CD, Vandenberg CJ, Ho G-Y*, et al.* Acquired RAD51CPromoter Methylation Loss Causes PARP Inhibitor Resistance in High-Grade Serous Ovarian Carcinoma. *Cancer Research* 2021;**81**(18):4709-22 doi 10.1158/0008-5472.Can-21-0774.

2. Kondrashova O, Topp M, Nesic K, Lieschke E, Ho G-Y, Harrell MI*, et al.* Methylation of all BRCA1 copies predicts response to the PARP inhibitor rucaparib in ovarian carcinoma. *Nature communications* 2018;**9**(1):3970.

3. Bedő J, Di Stefano L, Papenfuss AT. Unifying package managers, workflow engines, and containers: Computational reproducibility with BioNix. *GigaScience* 2020;**9**(11):giaa121.

4. Frankish A, Diekhans M, Jungreis I, Lagarde J, Loveland JE, Mudge JM*, et al.* GENCODE 2021. *Nucleic acids research* 2021;**49**(D1):D916-D23.

5. Sahlin K. Strobealign: flexible seed size enables ultra-fast and accurate read alignment. *Genome Biology* 2022;**23**(1):260.

6. Cooke DP, Wedge DC, Lunter G. A unified haplotype-based method for accurate and comprehensive variant calling. *Nature Biotechnology* 2021;**39**(7):885-92 doi 10.1038/s41587-021-00861-3.

7. Harrison PW, Amode MR, Austine-Orimoloye O, Azov AG, Barba M, Barnes I*, et al.* Ensembl 2024. *Nucleic acids research* 2024;**52**(D1):D891-D9.

8. Liu X, Jian X, Boerwinkle E. dbNSFP: a lightweight database of human nonsynonymous SNPs and their functional predictions. *Human mutation* 2011;**32**(8):894-9.

9. Cingolani P, Platts A, Wang LL, Coon M, Nguyen T, Wang L*, et al.* A program for annotating and predicting the effects of single nucleotide polymorphisms, SnpEff: SNPs in the genome of Drosophila melanogaster strain w1118; iso-2; iso-3. *fly* 2012;**6**(2):80-92.

10. Shen R, Seshan VE. FACETS: allele-specific copy number and clonal heterogeneity analysis tool for high-throughput DNA sequencing. *Nucleic acids research* 2016;**44**(16):e131-e.

11. Marquard AM, Eklund AC, Joshi T, Krystanek M, Favero F, Wang ZC, *et al.* Pan-cancer analysis of genomic scar signatures associated with homologous recombination deficiency suggests novel indications for existing cancer drugs. *Biomark. Res.* 2015;**3**:9

12. Bankhead P, Loughrey MB, Fernández JA, Dombrowski Y, McArt DG, Dunne PD*, et al.* QuPath: Open source software for digital pathology image analysis. *Scientific reports* 2017;**7**(1):1-7.

13. Schmidt U, Weigert M, Broaddus C, Myers G. Cell detection with star-convex polygons. 2018. Springer. p 265-73.

14. Team RC. R: A language and environment for statistical computing. Vienna, Austria. : R Foundation for Statistical Computing; 2023.

15. Wickham H, Averick M, Bryan J, Chang W, McGowan LDA, François R, *et al*. Welcome to the Tidyverse. *Journal of Open Source Software,* 2019 **4**(43) doi https://doi.org/10.21105/joss.01686.

16. Gu Z. Complex heatmap visualization. *iMeta* 2022;**1**(3):e43 doi https://doi.org/10.1002/imt2.43.
